# Zeaxanthin Suppresses Azoxymethane/Dextran Sodium Sulfate–Induced Colorectal Cancer in Mice by Inhibiting Inflammation and Proliferation

**DOI:** 10.5152/tjg.2026.25661

**Published:** 2026-07-30

**Authors:** Mengxiang Yang, Yuejun Han, Ting Jin, Jianping Wang

**Affiliations:** 1 Department of Colorectal Surgery, Affiliated Jinhua Hospital, Zhejiang University School of Medicine, Zhejiang, China; 2 Department of Gastroenterology, Affiliated Jinhua Hospital, Zhejiang University School of Medicine, Zhejiang, China

**Keywords:** Colorectal cancer, inflammation, proliferation, zeaxanthin

## Abstract

**Background/Aims:**

The treatment of colorectal cancer (CRC) remains a challenge in clinical research. Zeaxanthin (ZEA) has demonstrated substantial anti-inflammatory and anticancer effects. However, its preventive effects and underlying mechanisms in CRC remain elusive. Therefore, this study investigated the preventive effects and 10 molecular mechanisms of ZEA in CRC.

**Materials and Methods:**

The effects of ZEA on tumor formation, inflammatory responses, and cell proliferation were investigated in an azoxymethane (AOM)/dextran sodium sulfate (DSS)–induced mouse model of CRC. Furthermore, the anti-inflammatory and antiproliferative mechanisms of ZEA were evaluated in vitro using lipopolysaccharide (LPS)-stimulated RAW264.7 macrophages and CRC cell lines (HCT116 and SW620).

**Results:**

Treatment with 50 mg/kg ZEA significantly inhibited AOM/DSS–induced CRC progression. Furthermore, treatment with ZEA reduced tumor formation in the colonic lining, increased body weight, enhanced colorectal length, improved overall survival rate, and attenuated pathological changes in mice. Moreover, ZEA significantly reduced proinflammatory cytokines (tumor necrosis factor-α and interleukin-1β) and Ki-67 expression levels. Mechanistically, ZEA mitigated inflammation in LPS-stimulated RAW264.7 macrophages by suppressing the nucleotide-binding oligomerization domain-containing protein 1 (NOD1)/receptor-interacting protein 2 kinase (RIP2)/nuclear factor-κB (NF-κB) signaling pathway. In addition, it suppressed proliferation of HCT116 and SW620 cells by inhibiting the signaling pathway of PI3K/AKT/MYC.

**Conclusion:**

ZEA exerted significant anti-CRC effects, including anti-inflammatory and antiproliferative actions through multitarget mechanisms, such as modulation of the NOD1/RIP2/NF-κB and PI3K/AKT/MYC signaling pathways. These findings suggest that ZEA is a promising natural chemopreventive agent for the prevention and treatment of CRC.

**Main Points**

- Zeaxanthin significantly prevents colorectal carcinogenesis in vivo.
- The chemopreventive effect is mediated through anti-inflammatory and antiproliferative actions.
- Zeaxanthin represents a promising natural candidate for CRC intervention.

## Introduction

Colorectal cancer (CRC) is a gastrointestinal malignancy with high incidence and mortality rates, both of which have increased in recent years.^1^^,^^2^ Currently, the primary clinical interventions for CRC are chemotherapy and surgery.^3^^,^^4^ However, despite their efficacy, commonly used chemopreventive drugs such as 5-fluorouracil, cisplatin, and oxaliplatin are often associated with drug resistance, low response rates, and severe side effects, which limit their overall chemopreventive efficacy and compromise patients’ quality of life. The high cost of these agents imposes a substantial economic burden on patients and healthcare systems.^3^^,^^5^^,^^6^ Natural compounds are an important source of new drugs, drug leads, and chemical agents, and more than half of anticancer drugs are derived from natural sources. Such natural products are highly effective, relatively inexpensive, and generally associated with off-target adverse effects than conventional therapies.^7^^,^^8^ Therefore, the identification and development of effective and safe alternative natural compounds are critical for the prevention and treatment of CRC.

Zeaxanthin (ZEA) is a natural carotenoid widely found in edible seaweeds.^9^^,^^10^ It exhibits various beneficial properties, such as anticancer, antioxidant, anti-inflammatory, and neuroprotective activities. Furthermore, ZEA has been shown to exert antitumor effects by stimulating apoptosis through ROS-regulated MAPK and AKT signaling pathways in human gastric cancer cells.^11^^,^^12^ Several pre-clinical and clinical studies have demonstrated the safety of ZEA.^13^^,^^14^ However, its effects and underlying mechanisms in CRC remain poorly understood and warrant further investigation.

The current study elucidated the anticancer effects of ZEA using an azoxymethane (AOM)/dextran sodium sulfate (DSS)–induced CRC mouse model. Furthermore, the underlying anti-tumor mechanisms of ZEA were evaluated in CRC cells. The findings provide a scientific basis for the further development of ZEA for the prevention and treatment of CRC.

## Materials and Methods

The study was conducted between March 2024 and December 2025 and included the establishment of an AOM/DSS–induced CRC mouse model, ZEA intervention, sample collection, and subsequent histological, molecular, and transcriptomic analyses.

### Animal Models

For in vivo assays, male C57BL/6 mice (age = 8-10 weeks) were housed in a specific pathogen-free facility under controlled conditions (temperature, 22 ± 2°C; relative humidity, 50 ± 10%; 12-hour light/12-hour dark cycle) with ad libitum access to chow and water. The study was approved by the Ethics Committee of Affiliated Jinhua Hospital, Zhejiang University School of Medicine (approval number AL- JHYY202423. Date: March 14, 2024). During the preparation of this manuscript, the authors used ChatGPT solely for language polishing and improving readability.

The CRC model was established as previously described.^15^ Briefly, mice received ZEA (50 mg/kg)by oral gavage every 3 days. On day 8, AOM (10 mg/kg; Sigma) was administered intraperitoneally (i.p.). After 5 days of regular chow and water, the AOM-treated mice received 1.5% DSS (35 000-50 000 kDa molecular weight, MP Biomedicals Inc. Solon, OH, USA) in drinking water for another 5 days. Then, the mice received regular water for 14 days and were subjected to 2 additional DSS treatment cycles. Body weight was recorded weekly. After 85 days of ZEA administration, all mice (10 per group) were euthanizedvia cervical dislocation, and macroscopic tumors were counted and measured using calipers.

### Hematoxylin and Eosin Staining

The tissue sections were deparaffinized, rehydrated, and stained with hematoxylin to visualize nuclei in blue or purple. The sections were then stained with eosin to visualize the cytoplasm and extracellular components in pink. The sections were then dehydrated, cleared, mounted, and examined using a light microscope. Paraffin-embedded tumor sections (4 μm) were stained with hematoxylin and eosin (H&E) and independently evaluated by 2 experienced pathologists blinded to the experimental groups. Histological scoring was performed using a semiquantitative system that assessed the following parameters: tumor differentiation (well differentiated, 1; moderately differentiated, 2; poorly differentiated, 3), nuclear pleomorphism (absent, 0; mild, 1; moderate, 2; marked, 3), mitotic activity (0, <5; 1, 5-10; 2, 11-20; 3, >20 per 10 high-power fields), and the extent of necrosis (absent, 0; <10%, 1; 10-30%, 2; >30%, 3). A composite histologic score ranging from 1 to 12 was calculated for each sample, with higher scores indicating more aggressive histologic features. In cases of disagreement, a consensus score was reached through joint reevaluation.

### Enzyme-Linked Immunosorbent Assay

Lipopolysaccharide (LPS)-stimulated mouse serum or cell supernatants were collected to determine interleukin-6 (IL-6) and IL-1β levels using the mouse tumor necrosis factor-α (TNF-α) and IL-1β ELISA kits (MultiSciences Biotech Co., Hangzhou, China) according to the manufacturer’s instructions. All experiments were performed 3 times, and cytokine levels were expressed as pg/mL.

### Western Blotting Analysis

Radioimmunoprecipitation assay lysis buffer (Solarbio, Beijing, China) was used to lyse treated cells and extract total protein. The extracted protein was quantifiedusing a BCA Protein Assay Kit (Beyotime, Shanghai, China). Equal amounts of protein were separated by 10% sodium dodecyl sulfate-polyacrylamide gel electrophoresis, and transferred to polyvinylidene difluoride membranes (0.22 μm, Millipore,Billerica, CA, USA), which were then blocked with 5% skimmed milk for 1 hour at room temperature and incubated overnight at 4°C with primary antibodies against nucleotide-binding oligomerization domain-containing protein 1 (NOD1) (1:1000, Cell Signaling Technology, Danvers, MA, USA), nuclear factor-κB (NF-κB) and p-NF-κB (1:1000, Cell Signaling Technology), p-RIP (1:1000, Invitrogen, CA, USA), c-MYC (1:1000, Abcam, MA, USA), PI3K and p-PI3K (1:1000, Cell Signaling Technology), AKT and p-AKT (1:1000, Proteintech, Wuhan, China), and GAPDH (1:1000, Proteintech). The membranes were then incubated with horseradish peroxidase-labeled secondary antibodies (1:2000, Beyotime, Shanghai, China) for 1 hour at ambient temperature. Protein bands were detected using an ECL detection kit (Fdbio, Hangzhou, China) and imagedusing the ChemiDoc XRS+ system (Bio-Rad, Hercules, CA, USA).

### Immunohistochemistry

Paraffin-embedded tissues were sectioned into 4- to 5-μm slices, baked, dewaxed, hydrated, subjected to antigen retrieval by dropwise addition of 3% H_2_O_2_, occluded for 30 minutes with 5% bovine serum albumin, and treated overnight with the primary antibody against Ki-67 (Proteintech, 1:200) and MYC (Proteintech, 1:200) at 4°C. The sections were then washed, incubated with the appropriate biotinylated secondary antibody for 1 hour, treated with 3,3′-diaminobenzidine reagent for color development, and examined under a light microscope. Finally, the sections were rinsed with ddH_2_O to terminate hematoxylin staining. The integrated optical density (IOD) value and area (corresponding to the sample area at 200 times the field of view) were measured by using Image-Pro Plus 6.0, and the IOD/area ratio was calculated as a semiquantitative analysis index.

### Cell Cultures and Transfection

The murine macrophage cell line RAW264.7 and human CRC cell lines HCT116 and SW620 were acquired from the Shanghai Chinese Academy of Sciences. All cells were cultured separately in DMEM, M5A, and L15 medium supplemented with 10% fetal bovine serum (FBS, Senbeijia, Nanjing, China), 100 μg/mL of streptomycin and 100 U/mL of penicillin at 37°C in a humidified atmosphere containing 5% CO_2_. The nucleotide sequences of the negative control and siRNA of c-MYC were obtained from Tsingke Biotechnology Co., Ltd. Cell transfection was then performed using Lipofectamine 2000 (Invitrogen) according to the manufacturer's instructions.

### Quantitative Real-time Polymerase Chain Reaction

Total RNA was extracted from tissues or cultured cells using TRIzol reagent and reverse transcribed into complementary DNAvia the Quantscript RT kit (Takara, Tokyo, Japan). Then, quantitative reverse transcription polymerase chain reaction (qRT-PCR) was then performed using the TB Green Fast PCR Kit (TaKaRa) on the Lightcycler® 480II qRT-PCR System. Primer sequences are detailed in Supplementary Table 1.

### CCK-8 Analysis

Cells (1 × 10^4^/well) were plated into 96-well plates and incubated in complete medium (100 μL) at 37°C. Then, CCK-8 solution (10 μL) was added to each well. After gentle shaking at 37°C for 1 hour, absorbance was measured at 450 nm using a Synergy HT Multi-Mode Microplate Reader (Bio-Tek, Winooski, VT, USA).

### Colony Formation Analysis

Cells (1000/well) were plated in 6-well plates and incubated at 37°C for 10-15 days. When visible colonies were formed, the cells were fixed with 4% paraformaldehyde (Fdbio) for 15 minutes and stained with Crystal Violet Staining Solution (Beyotime, Shanghai, China) for 20 minutes. Lastly, cell colonies were counted and evaluated.

### RNA-Seq Analysis

To characterize the transcriptomic alterations induced by ZEA, HCT116 human colorectal carcinoma cells were treated with either ZEA or phosphate-buffered saline (PBS) as a vehicle control. Total RNA was extracted from 3 independent biological replicates per group, and libraries were constructed using a standard poly(A)-enrichment protocol. Sequencing was performed on an Illumina platform (e.g., NovaSeq 6000), generating paired-end 150-bp reads. After quality control using FastQC and adapter trimming using Trimmomatic, clean reads were aligned to the human reference genome (GRCh38) using HISAT2. Transcript abundance was quantified as FPKM (fragments per kilobase of transcript per million mapped fragments), which served as a normalized measure of gene expression for visualization and comparative analyses. For differential expression analysis, raw gene counts were obtained using featureCounts and analyzed using the DESeq2 package in R (version 4.2.0). Differentially expressed genes (DEGs) between the ZEA and PBS groups were identified using a threshold of |log₂(fold change)| > 2 and an adjusted *P*-value (Benjamini–Hochberg correction) < .05. Subsequently, to gain insight into the biological pathways perturbed by treatment with ZEA, Kyoto Encyclopedia of Genes and Genomes (KEGG) pathway enrichment analysis of the DEGs was performed with the clusterProfiler package, using the human annotation database org.Hs.eg.db. Enriched pathways with a false discovery rate < 0.05 were considered statistically significant, and the results were visualized using dot plots and bar charts.

### Statistical Analysis

Each experiment was performed at least 3 times. GraphPad Prism 7 (GraphPad Software; San Diego, CA, USA) was used for graph generation and data analysis. The results are depicted as mean ± SD. Student’s *t*-test was used to analyze differences between the 2 groups, whereas 1-way analysis of variance followed by individual comparisons with Tukey’s test was used for comparisons among more than 2 groups. Survival distributions and overall survival (OS) rates were estimated using the Kaplan–Meier method, and differences between survival rates were assessed using the log-rank test. A 2-tailed *P* < .05 was considered statistically significant.

## Results

### ZEA Alleviated AOM/DSS–Induced CRC in Mice

The antitumor activity of ZEA was assessed in mice injected intraperitoneally with AOM (a potent inducer of cancer and precancerous lesions), followed by repeated administration of DSS to induce colorectal tumors. During AOM/DSS treatment, mice received oral ZEA (50 mg/kg) 3 times weekly for 12 weeks (Supplementary Figure 1). Compared with the control mice, mice in the AOM/DSS group exhibited a significant 17.27% decrease in body weight (Figure 1A); however, this effect was attenuated by treatment with ZEA. Furthermore, the Kaplan–Meier survival curve (Supplementary Figure 1) showed that ZEA improved the survival rate of mice with CRC At the end of the study, survival rates were 80% in the AOM/DSS group and 100% in the ZEA-treated group. Moreover, the incidence of CRC was evaluated by assessing tumor number and distribution. Compared with the AOM/DSS group, treatment with ZEA markedly suppressed tumorigenesis, reduced tumor burden, and attenuated colon length shortening (*P < .05) (Figure 1B).

Histological analysis revealed no malignant transformation or obvious pathological changes in the control and the ZEA single-treatment group of mice. However, mice in the AOM/DSS group exhibited characteristic features of malignant transformation, including epithelial erosion, crypt destruction, glandular hyperplasia, and significant inflammatory responses. Nevertheless, ZEA treatment markedly alleviated the colorectal inflammatory and tumor pathological changes induced by AOM/DSS (Figure 1C). These findings indicated that ZEA suppressed carcinogenesis and tumor progression in the AOM/DSS mouse group.

### ZEA Inhibited Proinflammatory Cytokines by Suppressing the NOD1/RIP2/NF-κB Signaling Pathway

Chronic inflammation is a key factor in CRC progression and promotes the activation of effector T cells and macrophages, leading to the release of various cytokines, such as TNF-α and IL-1β. These cytokines promote CRC onset and progression by suppressing tumor suppressor genes, inducing DNA damage and upregulating antiapoptotic protein genes.^2^. This study also evaluated the levels of inflammation-related genes by qRT-PCR and ELISA. The data indicated that levels of IL-1β and TNF-α were increased in the AOM/DSS cohort compared to the control cohort. However, ZEA treatment markedly reduced TNF-α and IL-1β by 21.36% and 50.88%, respectively (Supplementary Figure 2).

Moreover, the potential mechanisms underlying the anti-inflammatory effects of ZEA were investigated. Tissue-resident macrophages are key promoters of the inflammatory response in various diseases, including CRC.^16^ Therefore, the effects of ZEA on the levels of related inflammatory factors in LPS-stimulated RAW 264.7 macrophages were evaluated. First, the cytotoxicity assay demonstrated that ZEA (10-100 µM) was not cytotoxic to RAW264.7 cells (Supplementary Figure 3). Then, an in vitro inflammation model was developed by stimulating RAW264.7 cells with LPS (1 μg/mL), followed by treatment with ZEA (10 μM). After treatment, both cell lysates and culture supernatants were collected to measure inflammatory cytokine levels by ELISA and qRT-PCR. LPS stimulation increased TNF-α and IL-1β levels by 5.33 fold and 2.82 fold, respectively, compared with the control group, whereas treatment with ZEA had the opposite effect (Figure 2A-2B). These results suggested that ZEA suppresses LPS-induced inflammatory responses.

Studies suggest that inflammatory responses are regulated by NOD1 and pattern recognition receptors (PRRs). An intracellular PRR in host cells initiates these responses by associating with receptor-interacting protein 2 kinase (RIP2), thus activating the downstream NF-κB signaling pathway.^17^ Here, the expression of inflammation-related proteins (NOD1, RIP2, and NF-κB) was evaluated by Western blotting. LPS markedly increased the expression of NOD1, p-RIP2, and p-NF-κB compared with the control group, suggesting activation of the NOD1/RIP2/NF-κB signaling pathway. Moreover, ZEA co-treatment had the opposite effect, suggesting that it significantly inhibited the NOD1/RIP2/NF-κB signaling pathway (Figure 2C). These results suggested that ZEA may alleviate intestinal inflammation by inhibiting the NOD1/RIP2/NF-κB signaling pathway.

### ZEA Inhibited the Proliferation of CRC Cells

Cancer is characterized by the aberrant regulation of proliferation and perpetual replication, resulting in unregulated cellular growth.^18^ Ki-67 is a cell proliferation marker primarily used to assess cell proliferative activity. This study demonstrated that AOM/DSS treatment increased the number of Ki-67-positive cells compared with the control group, whereas treatment with ZEA markedly reduced the number of Ki-67-positive cells (Figure 3A), suggesting that ZEA suppressed AOM/DSS-induced tumor growth. Moreover, the direct effects of ZEA on the viability and proliferation of CRC cell lines were evaluated. ZEA markedly inhibited the viability of HCT116 and SW620 cells in a concentration-dependent manner at concentrations ranging from 5 to 20 μM (Figure 3B). In addition, a colony formation assay was performed to evaluate the inhibitory effects of ZEA on the proliferative capacity of HCT116 and SW620 cells. After 14 days of treatment with ZEA, the number of colonies was significantly reduced by 56.84% in HCT116 cells and 43.75% in SW620 cells (Figure 3C). These findings indicated that ZEA markedly inhibited the proliferation of CRC cells.

### ZEA Inhibited the PI3K/AKT/MYC Signaling Pathway

To investigate the mechanism by which ZEA suppresses CRC cell proliferation, RNA-seq was performed on HCT116 cells treated with either ZEA or PBS. KEGG pathway enrichment analysis revealed significant enrichment of the PI3K/AKT signaling pathway (Figure 4A). This signaling pathway has been implicated in apoptosis, tumor proliferation, and metastasis.^5,6^ Therefore, the effects of ZEA on the PI3K/AKT signaling pathway were evaluated by Western blotting, which demonstrated that treatment with ZEA reduced the p-PI3K/PI3K and p- AKT/AKT ratios (Figure 4B). These findings suggested that ZEA may inhibit CRC cell proliferation, at least in part, by modulating the PI3K/AKT signaling pathway. To investigate how ZEA regulates this pathway, the RNA-seq data were analyzed to identify key genes variably expressed in the PI3K/AKT signaling pathway between ZEA-treated and control groups. Moreover, these genes were validated by qRT-PCR, which demonstrated that MYC mRNA expression was most markedly downregulated following ZEA treatment (Figure 5A). In addition, HCT116 and SW620 cells validated that therapy using ZEA reduced MYC protein levels (Figure 5B). Furthermore, the immunohistochemical assay showed that AOM/DSS treatment substantially increased the number of MYC-positive cells compared with the control group, whereas treatment with ZEA effectively reversed this increase (Figure 5C). These findings suggested that MYC is a downstream effector of the PI3K/AKT signaling pathway.

To evaluate whether MYC is crucial for ZEA-mediated modulation of CRC cell proliferation, MYC was silenced in HCT116 and SW620 cells (Supplementary Figure 4A). The CCK-8 assay demonstrated that MYC downregulation substantially inhibited the proliferation of HCT116 and SW620 cells (Supplementary Figure 4B). The colony formation assay demonstrated that MYC downregulation significantly suppressed the proliferative capacity of HCT116 and SW620 cells (Supplementary Figure 4C). These findings demonstrate that MYC downregulation suppresses CRC cell proliferation. These results suggest that ZEA inhibits CRC proliferation through the PI3K/AKT/MYC signaling pathway.

## Discussion

Epidemiological studies have identified CRC as the third most prevalent cancer worldwide and the second leading cause of cancer-related mortality.^1^^,^^2^ Furthermore, the incidence and mortality rates of CRC are steadily increasing.^1^^,^^19^ Despite substantial advances in biological treatments, immunotherapies, and modern drug discovery, chemotherapy continues to be the fundamental approach for CRC treatment in clinical practice.^3^^,^^4^ However, the chemopreventive efficacy of chemotherapy is often limited by its inherent cytotoxicity, the progression of treatment resistance, and substantial patient morbidity.^3^ These limitations underscore the need to develop low-toxicity treatment agents and effective treatment strategies for CRC. In the present study, the authors systematically analyzed the preventive effects of ZEA against CRC and the underlying molecular mechanisms. Using an AOM/DSS–induced CRC mouse model, the authors demonstrated that ZEA treatment, particularly at the dose of 50 mg/kg, markedly inhibited CRC progression in vivo. ZEA effectively alleviated colonic pathological lesions, reduced tumor burden, restored body weight and colorectal length, and improved survival in AOM/DSS-treated mice. Meanwhile, ZEA exerted potent anti-inflammatory effects by reducing the expression of proinflammatory cytokines (TNF-α and IL-1β) and suppressing excessive cell proliferation, as evidenced by decreased Ki-67 expression. Further mechanistic investigations demonstrated that ZEA inhibited inflammatory responses in LPS-stimulated RAW264.7 macrophages by suppressing the NOD1/RIP2/NF-κB signaling pathway. Additionally, ZEA inhibited the proliferation of HCT116 and SW620 CRC cells by inactivating the PI3K/AKT/MYC signaling pathway. Collectively, these findings demonstrate that ZEA exerts anti-CRC effects through dual anti-inflammatory and antiproliferative actions mediated by regulation of 2 core signaling pathways.

Therefore, natural compounds are considered promising candidates for the prevention and treatment of CRC because of their diverse chemical structures, improved chemopreventive efficacy, and improved safety profile.^7^^,^^8^ Seaweed-derived compounds have attracted considerable attention because of their historical application in traditional Asian medicine and their association with a 50% reduction in CRC risk.^20^ ZEA is a carotenoid commonly found in edible seaweeds. It has been comprehensively studied for its chemopreventive potential in various diseases, specifically ocular disorders.^9^^,^^10^^,^^21^^-^^23^ Recent studies have demonstrated that ZEA exhibits anticancer activity in multiple cancer types, such as breast, bladder, lung, gastric, and skin cancers.^24^ However, specific mechanisms and chemopreventive potential of ZEA in CRC remain poorly understood. Therefore, this study investigated the effects of ZEA on CRC progression and the underlying molecular mechanisms.

This study used an AOM/DSS-induced CRC mouse model, which closely recapitulates the pathological progression of human CRC.^25^ The data indicated remarkable chemopreventive efficacy of treatment with ZEA in this model, substantially increasing survival rates while reducing characteristic disease symptoms, such as weight loss, tumor development, and colon shortening. Histopathological analysis demonstrated that therapy using ZEA successfully mitigated AOM/DSS–induced CRC, primarily by reducing epithelial erosion, crypt damage, and glandular hyperplasia. The results suggest that ZEA may substantially inhibit the progression of CRC and is a promising candidate for the prevention and treatment of CRC.

Chronic intestinal inflammation is considered to promote CRC progression and affects all phases of tumor formation, from initiation to metastasis.^26^ The inflammatory response in CRC is characterized by the upregulation of key mediators, including IL-1β and TNF-α.^27^ Furthermore, the anti-inflammatory properties of ZEA have been demonstrated in various inflammation-related diseases,^28^^,^^29^ where it inhibits the production of proinflammatory cytokines.^30^ In the present study, treatment with ZEA significantly reduced AOM/DSS–induced production of IL-1β and TNF-α. Moreover, the in vitro LPS-induced RAW264.7 macrophage model demonstrated that ZEA exerts anti-inflammatory effects by inhibiting the NOD1/RIP2/NF-κB signaling pathway, an essential factor for regulating inflammation.^17^ This finding is particularly relevant because NOD1 is associated with inflammatory reactions in CRC.^31^

Uncontrolled cell proliferation is a key hallmark of cancer.^18^ In the current study, in vivo analyses demonstrated that ZEA markedly reduced the number of Ki-67-positive cells in AOM/DSS-induced mice, indicating potent antiproliferative activity. The results were supported by in vitro experiments demonstrating that ZEA inhibited the proliferation of CRC cells. Mechanistic studies identified the signaling pathway of PI3K/AKT as a principal target of the antiproliferative effects of ZEA. This pathway regulates essential biological activities such as proliferation, survival, and apoptosis^32^ and is associated with CRC progression. The results of this study demonstrated that ZEA markedly inhibited the PI3K/AKT signaling pathway, as evidenced by reduced phosphorylation levels.

Subsequent downstream investigation showed that the effects of ZEA on cellular proliferation are mediated by the regulation of MYC (c-MYC) expression. Therefore, MYC, a key downstream effector of the PI3K/AKT signaling pathway and a proto-oncogene that regulates cell proliferation and metabolism,^33^^,^^34^ is a significant chemopreventive target in CRC. This study showed that treatment with ZEA reduced MYC expression, thereby inhibiting the CRC cell proliferation. These findings suggest that the antiproliferative effects of ZEA are mediated through the PI3K/AKT/MYC signaling pathway.

This study has several major strengths. The current study demonstrates the preventive effect of ZEA on CRC, providing new evidence supporting the application of natural products for the prevention and treatment of CRC. Furthermore, the authors demonstrate that ZEA exerts anti-inflammatory effects by inhibiting the NOD1/RIP2/NF-κB signaling pathway while simultaneously suppressing CRC cell proliferation through inhibition of the PI3K/AKT/MYC signaling pathway. This provides new insights into the multitarget anticancer effects of ZEA. Nevertheless, several limitations of the present study should be acknowledged. First, the use of a single dose of ZEA in vivo precludes the evaluation of dose–response relationships. Second, the absence of specific chemical pathway inhibitors restricts the depth of mechanistic validation. Furthermore, this was a preclinical study, and the pharmacokinetic characteristics, effective dosage, bioavailability, and preventive efficacy of ZEA in humans remain to be established. With respect to clinical safety, although ZEA has demonstrated a favorable safety profile in most clinical studies,^35^ mild gastrointestinal symptoms—including bloating and gastric discomfort, particularly under fasting conditions—have been reported in a small number of participants.^36^ Additionally, given the glucose-lowering effects of ZEA observed in animal models, it may theoretically enhance the effects of hypoglycemic agents, warranting caution with concomitant use.^37^ Notably, no clinical cases of such an interaction have been reported to date. Beyond safety considerations, ZEA is not the only natural compound with potential anti-CRC activity. Other naturally occurring compounds, such as matrine and aconitine, have also demonstrated anti-CRC effects through distinct mechanisms.^38^^,^^39^ However, similar to ZEA, most of these compounds face the common challenge of translating robust preclinical evidence into established clinical benefit.

In conclusion, this study provided evidence supporting the potential of ZEA as a chemopreventive agent for CRC. In vivo and in vitro findings demonstrated that ZEA effectively suppressed CRC progression through 2 mechanisms: (1) attenuation of intestinal inflammation by inhibition of the NOD1/RIP2/NF-κB signaling pathway and (2) inhibition of tumor cell proliferation through PI3K/AKT–mediated regulation of MYC expression. These findings support ZEA as a promising natural compound with substantial potential for chemopreventive applications in CRC. Future studies should investigate the molecular targets of ZEA and evaluate its chemopreventive effectiveness in clinical settings.

## Figures and Tables

**Figure 1.**
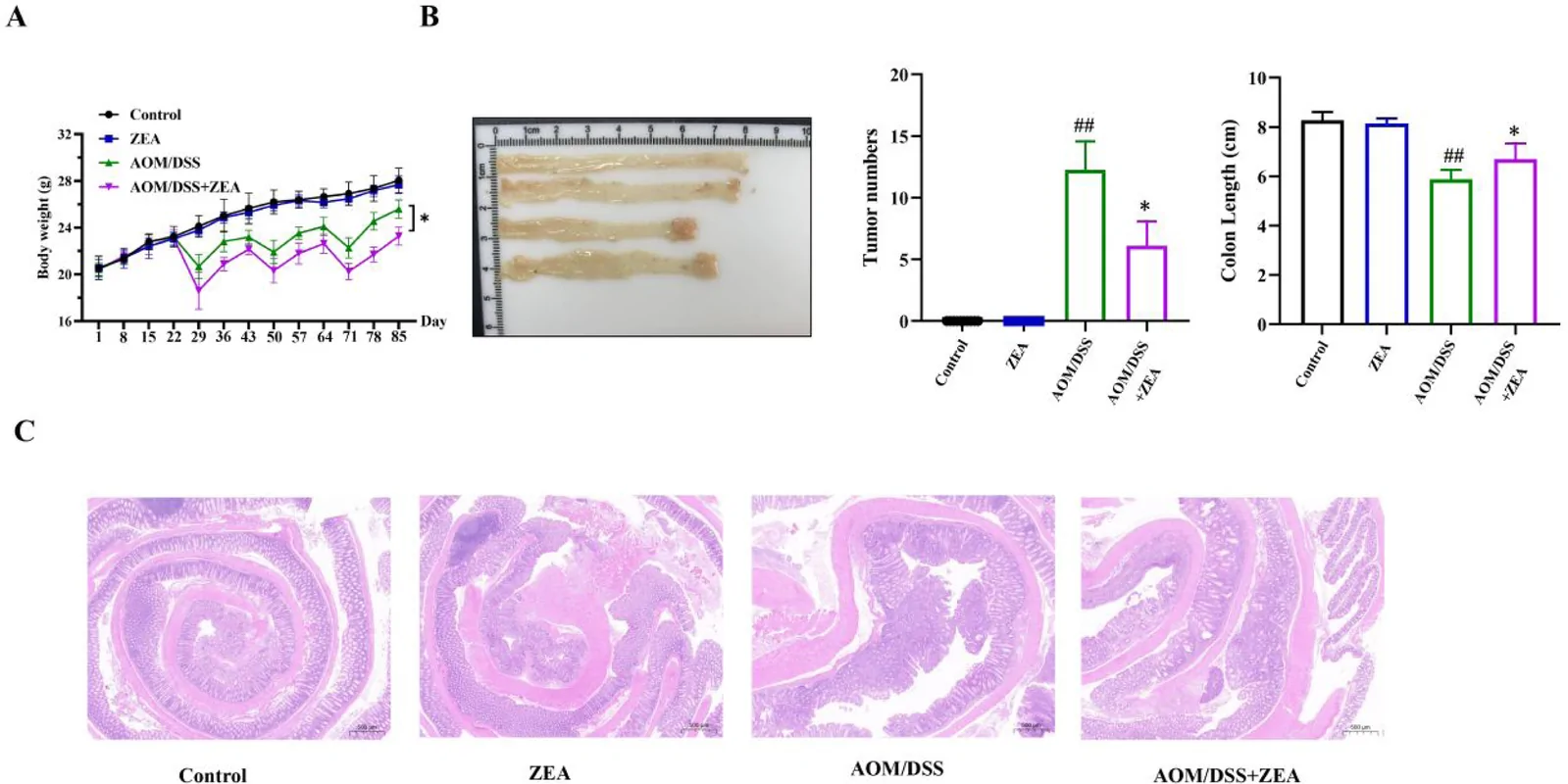
Zeaxanthin (ZEA) alleviates azoxymethane/dextran sodium sulfate (AOM/DSS)–induced colorectal tumorigenesis in mice. (A) Quantification of body weight of control and experimental mice. (B) Representative macroscopic images of the colon tumor region and corresponding quantification of tumor number and tumor length in control and experimental mice. (C) Hematoxylin and eosin–stained images of serial sections of the colon, along with quantitative analysis. n = 10. Data are presented as mean ± SD. *P < .05 compared with the AOM/DSS group. ^##^*P* < .01 compared with the control group.

**Figure 2.**
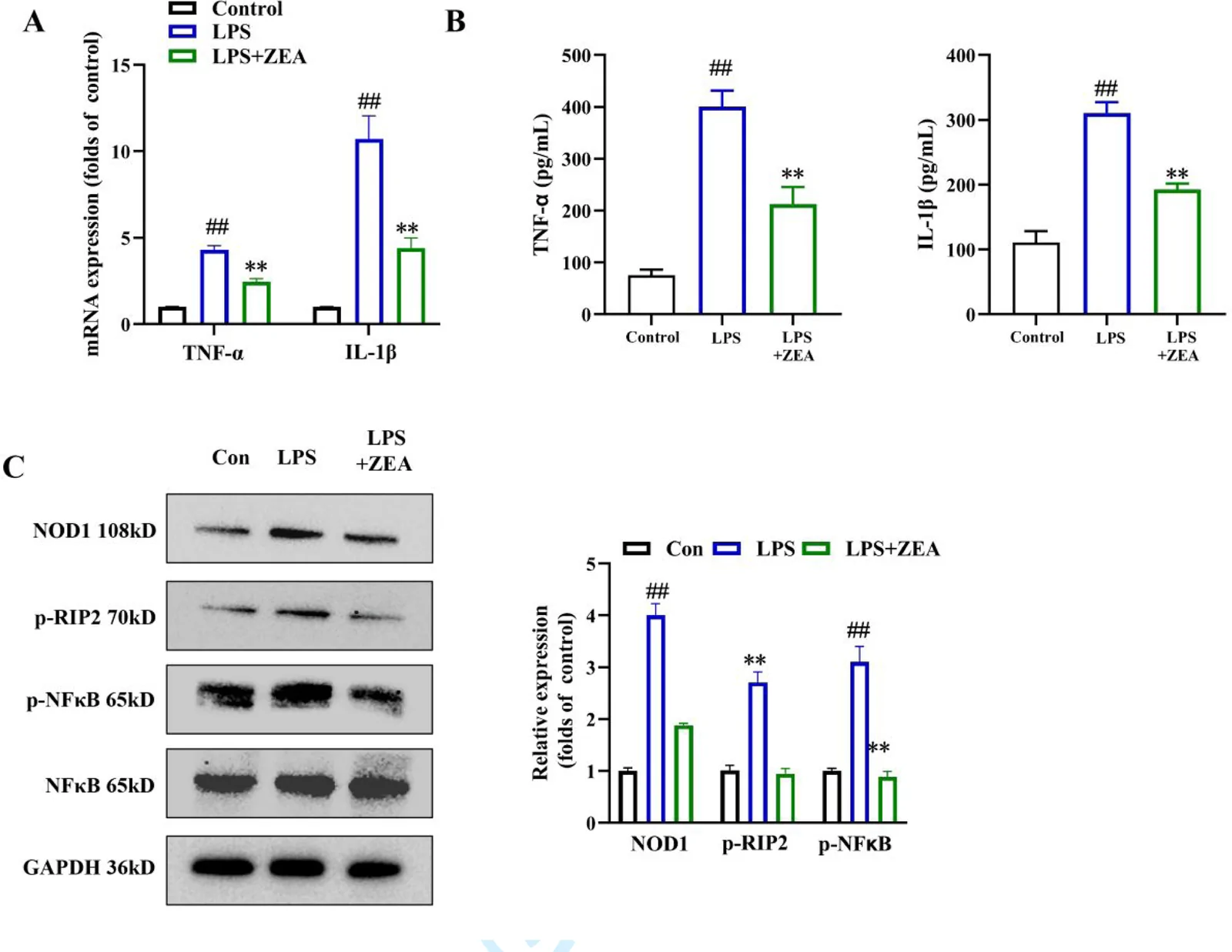
Zeaxanthin (ZEA) inhibits lipopolysaccharide (LPS)-induced inflammation in RAW264.7 cells. (A) Relative mRNA expression levels of TNFα and IL-1β by quantitative reverse transcription polymerase chain reaction (qRT-PCR). (B) TNF-α and IL-1β concentrations in culture supernatants were measured by enzyme-linked immunosorbent assay (ELISA). (C) The expression of NOD1, p-RIP2, p-NF-κBp65, and NF-κBp65 detected by Western blotting. NF-κB; nuclear factor-κB; NOD1, nucleotide-binding oligomerization domain-containing protein 1; RIP2, receptor-interacting protein 2 kinase.

**Figure 3.**
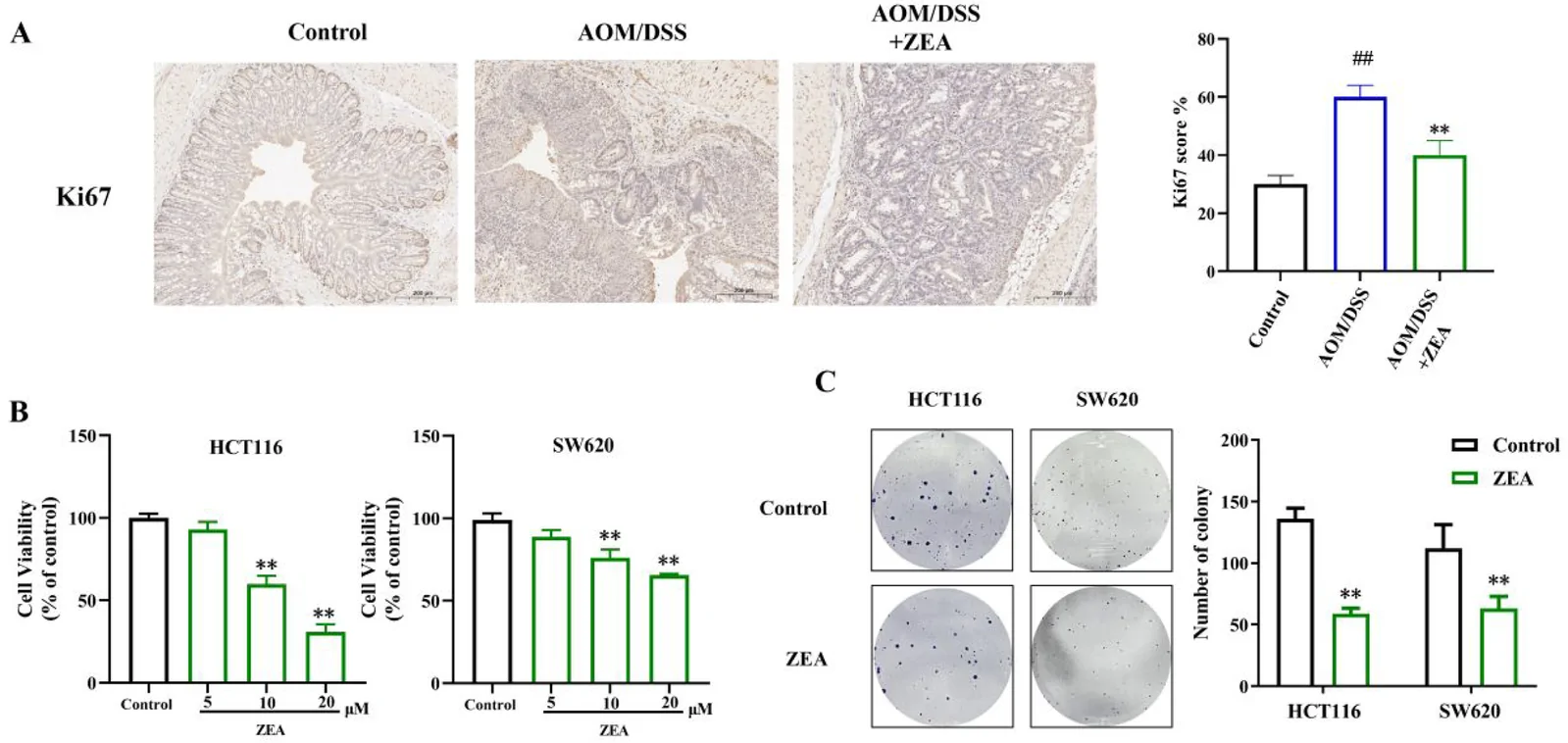
Zeaxanthin (ZEA) inhibited the proliferation of cancer cells. (A) Representative immunohistochemical images showing Ki-67 expression in tumor tissues from each experimental group of mice, along with quantitative analysis of Ki-67 expression. (B) Viability of HCT116 and SW620 cells cultured in the absence or presence of 5, 10, or 20 μM ZEA for 48 hours was assessed using the CCK-8 assay. (C) The influence of ZEA (10 μM) on colony formation. *P <.05, **P< .01 compared with the control group. ^#^*P* < .05*,* ^##^*P* < .01 compared with the azoxymethane/dextran sodium sulfate (AOM/DSS) group.

**Figure 4.**
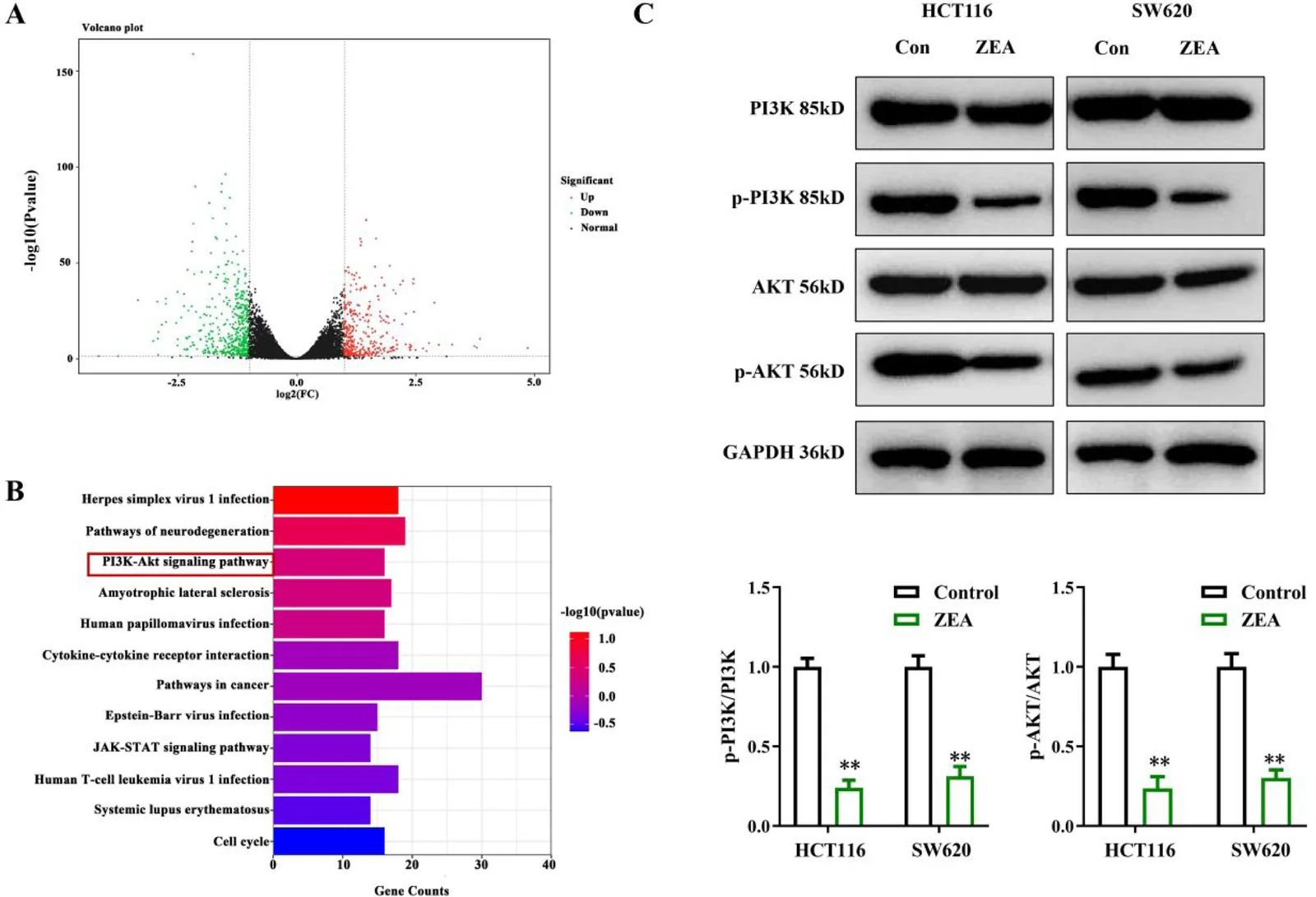
Zeaxanthin (ZEA) inhibited the proliferation of colorectal cancer (CRC) cellsvia the PI3K/AKT signaling pathway. (A) Kyoto Encyclopedia of Genes and Genomes pathway analysis for ZEA. Pathways are shown on the *y*-axis, whereas the enrichment factors are shown on the *x*-xis. (B) Western blot analysis for the expression of p-PI3K, PI3K, p-AKT, and AKT proteins.

**Figure 5.**
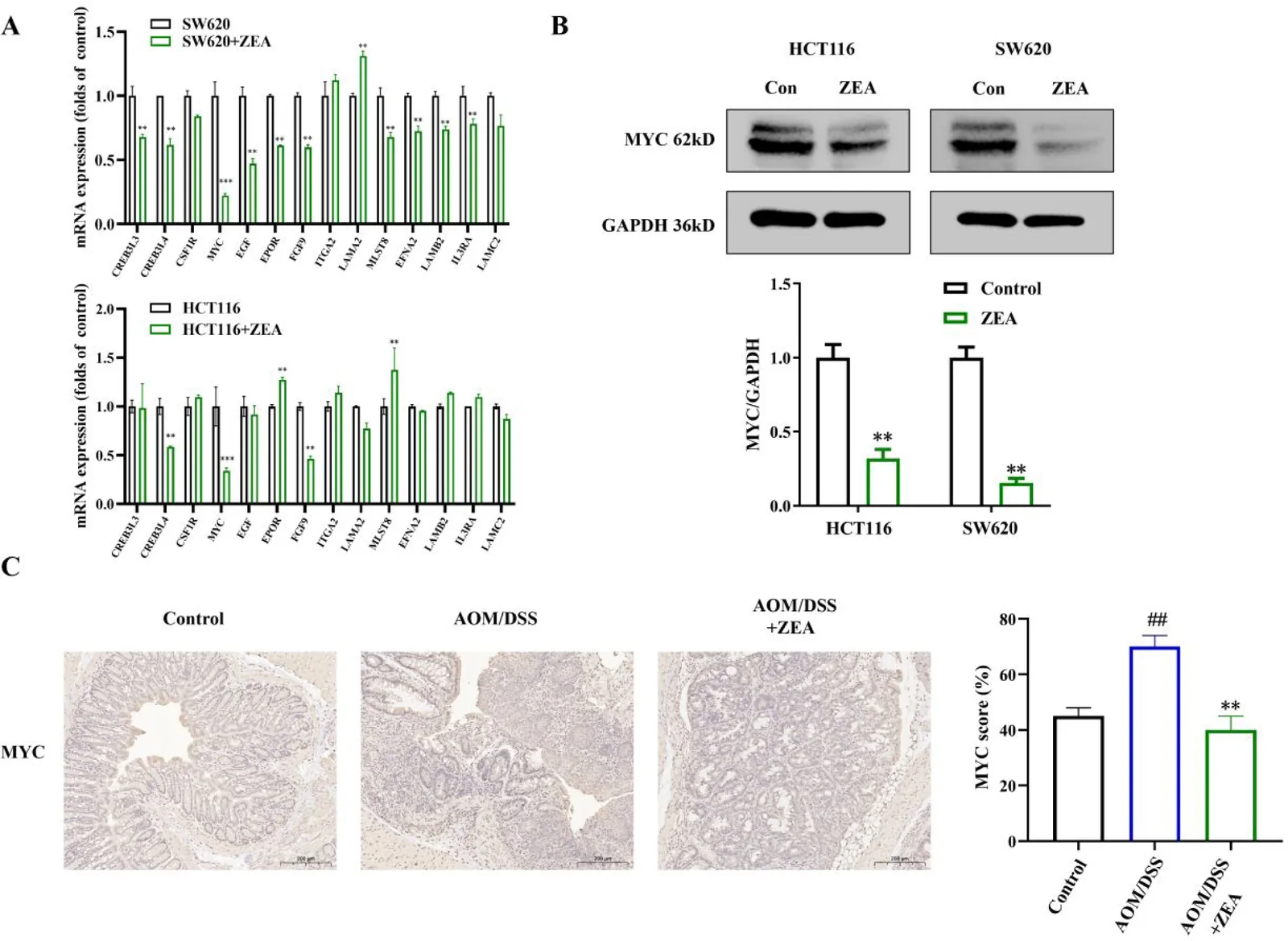
Zeaxanthin (ZEA) inhibited the expression of MYC. (A) Relative mRNA expression levels of PI3K/AKT–related genes. (B) The determination of the protein expression of MYC through the process of Western blotting. The expression levels of MYC were detected by Western blotting. GAPDH was used as a loading control. (C) Representative immunohistochemical (IHC) images illustrating MYC expression in tumors from each experimental group of mice. Quantitative analysis of MYC expression levels. *P< .05, **P < .01 compared with the control group. ^#^*P* < .05,^##^*P* < .01 compared with the azoxymethane/dextran sodium sulfate (AOM/DSS) group.

## Data Availability

The data that support the findings of this study are available on request from the corresponding author.
